# Dr. William B. Hart

**Published:** 1911-04

**Authors:** 


Dr. William B. Hart, University of Buffalo (1846) died at his home in Greenwood, Illinois, February 19, 1911, aged 99 years.

